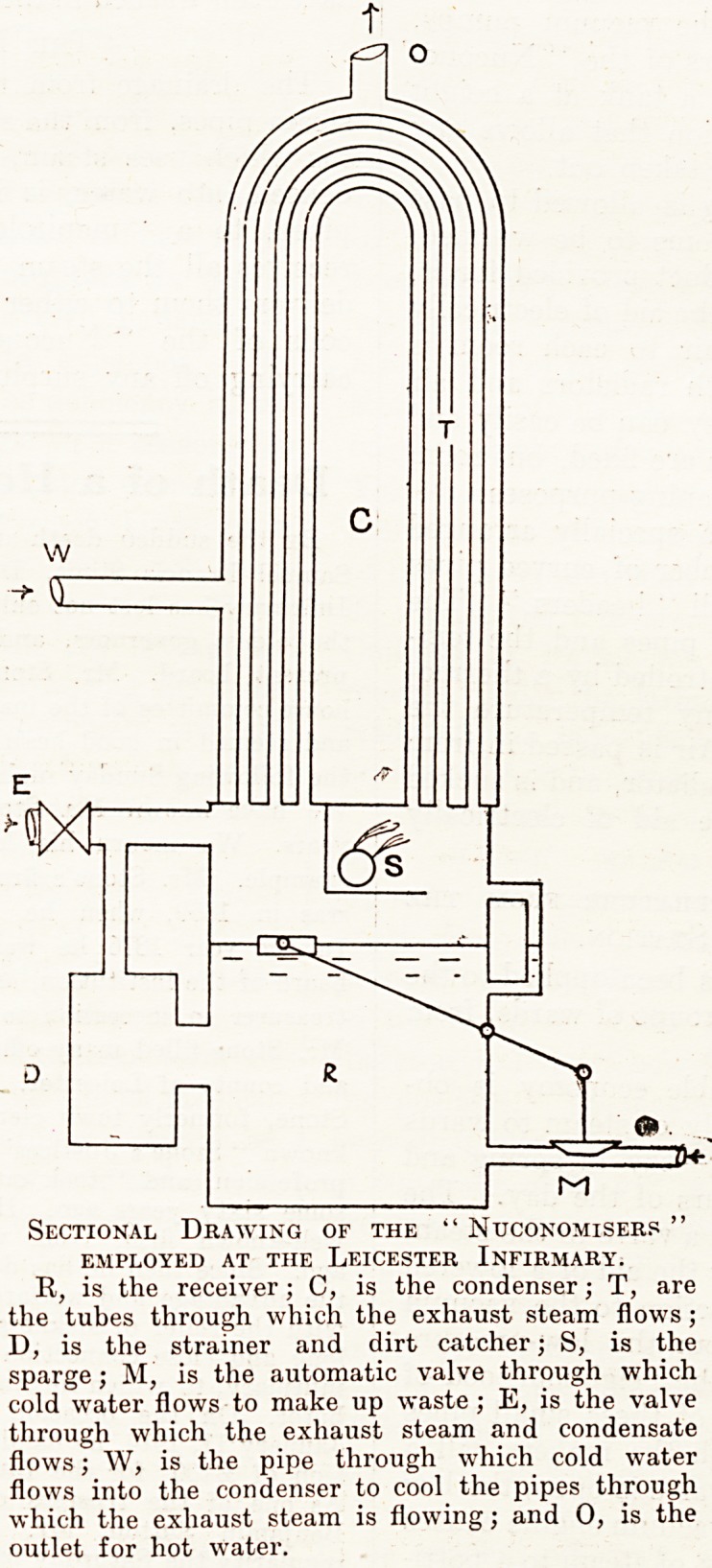# Leicester Infirmary: Heating by Steam below Atmospheric Pressure

**Published:** 1915-07-31

**Authors:** 


### THE HEATING OF HOSPITALS.

**[Note.—In a series of articles the writer proposes to describe the different systems of heating that are employed in hospitals. He will be pleased to answer any questions through "The Hospital," bearing upon the subject of heating or ventilation. Each system will be illustrated and described as exemplified at some hospital where it has been applied. In the first article the method by the use of low-pressure steam employed at Leicester Infirmary is dealt with.]**

### Leicester Infirmary.

### HEATING BY STEAM BELOW ATMOSPHERIC PRESSURE.

The officials at Leicester Infirmary claim, with legitimate pride, that while their hospital has been trebled in the last fifteen years, there has been no increase in the amount of the coal bill. Bearing in mind that the price of coal has risen during that period, this fact speaks very highly for the economies that have been carried out. Economy has been obtained entirely by utilising the heat contained in the hot water that previously was allowed to run to the drains, and in the steam that was allowed to discharge into the atmosphere.

In an article by the present writer which appeared in The Hospital some months back, it was mentioned that the greater portion of the heat required to be expended in boilers to raise steam was absorbed in converting the boiling water into steam at the same temperature. When the steam is allowed to escape to the atmosphere, the whole of this heat is wasted. At Leicester, all of the exhaust steam, and all of the water formed by the condensation of steam, is carried to a central heating station, where it is employed in raising the temperature of water for feeding the boilers, and for the service of the hospital.

### The Heating Plant.

The heating plant consists of two Lancashire boilers, one 9 ft. in diameter by 27 ft. long; the other 7 ft. in diameter by 25 ft. long.

One boiler is sufficient in summer, both boilers are required in winter. In the central heating station there are three apparatus called by the makers, "Nuconomisers." The apparatus is shown in section in the illustration. It is really a condenser, a receiver, and a calorifier in one. From the drawing it will be seen that it consists of a vertical cylinder in three parts. The lower portion is the receiver into which all the hot water is delivered. The level of the water in the receiver is also made to control an automatic supply valve, connected to the cold-water main. Cold water is allowed to run into the receiver, to make up any waste that occurs. In the upper part of the apparatus there are a number of tubes in the form of inverted U's. The exhaust steam is made to pass through the tubes; water, either from the water-main or from any other source available, being made to flow round the tubes, and to condense a portion of the steam. To complete the condensation of the steam, what the makers call a "sparge" is fixed in the middle portion of the apparatus, at the end of the tubes from which the condensed and uncondensed steam issues.

Sectional Drawing of the "Nuconomisers" employed at the Leicester Infirmary.
R, is the receiver; C, is the condenser; T, are the tubes through which the exhaust steam flows; D, is the strainer and dirt catcher; S, is the sparge; M, is the automatic valve through which cold water flows to make up waste; E, is the valve through which the exhaust steam and condensate flows; W, is the pipe through which cold water flows into the condenser to cool the pipes through which the exhaust steam is flowing; and O, is the outlet for hot water.

The sparge consists of a perforated tube, water being forced through the perforations in minute jets, and meeting the steam issuing from the ends of the tubes and condensing it. The condensed steam and the hot water fall together into the receiver. The feed pumps for the boiler, which are in duplicate to guard against breakdown, take their supply of water from the receiver, its temperature being usually in the neighbourhood of 200° F. As will be seen from the drawing also, there is a strainer and dirt catcher in the path of the condensed water, which cleanses the water before it reaches the receiver. It is also arranged to soften the cold water coming from the cold-water service by the introduction of softening fluids when it is necessary. An oil separator abstracts any oil that may be present from the steam before it reaches the "Nuconomiser." The domestic supply of hot water is taken from the receiver by the aid of pumps, in duplicate, which force it through the building.

The Heating of the Wards.

The heating of the wards is carried out partly on the "plenum" system and partly by means of radiators, in which steam below atmospheric pressure is employed. The steam from the boilers is taken through a reducing valve, by which its pressure is lowered to 1 lb. or 2 lb. above the atmosphere, to pipes leading to the radiators.

A return pipe from the radiators leads to two vacuum pumps. The radiators are connected between the steam supply pipe and the pipe leading to the vacuum pump, each radiator being controlled by a valve, by which more or less steam is allowed to enter. The steam expands in the radiators down to a temperature of about 180° F. The steam and condensed water from the radiators are sucked through the return pipes by the vacuum pumps, and are delivered to the receivers of the "Nuconomisers" after passing through a tank at a height above the central heating station that allows any air that may be present to be taken out.

Air from outside the building is allowed to pass over the radiators, into the rooms to be warmed, and is sucked out through a duct provided for it, usually near the floor line, by the aid of electrically driven fans. The supply of air to each room is controlled by a valve, and both radiators and air valves are so arranged that they can be easily and quickly cleaned. The radiators are fixed, but room is allowed behind them, for cleaning purposes.

The operating theatre has a specially arranged radiator. It consists of a number of curved pipes connected to what engineers call "headers." The quantity of steam entering the pipes and the temperature of the theatre are controlled by a thermostat, which can be set at any temperature the operating surgeon requires. Air is passed in from outside the building over the radiator, and is sucked out through the ducts by the aid of electrically driven fans.

The Control of the Temperature from the Central Heating Station.

A very interesting system has been applied to the control of the temperature in groups of wards, from the central heating station.

It is claimed that considerable economy is obtained by shutting off the supply of steam to wards upon which the sun is shining—say in spring and autumn—often for several hours of the day. The supply of steam is controlled by a valve in the steam pipe; the valve being moved by the aid of a lowered pressure produced by a connection to the vacuum engine. Small pipes lead from the low-pressure side of the vacuum engine to the neighbourhood of the different blocks. In each of these small pipes is placed a small valve, which the makers call a "switch." When the switch valve is open, the low pressure from that side of the vacuum engine causes the valve controlling the supply of steam to a particular block to close. When the temperature of the atmosphere lowers again, as it does during the afternoons of spring and autumn, closing the switch valve causes the steam valve at the entrance to the particular block to open, and steam to flow again into the radiators. The switch valves for the different blocks of buildings are fixed together on a switchboard in the central heating station.

Drying the Clothes in the Laundry.

Another economy has been obtained by the use of the exhaust steam from the engine which drives the laundry, to provide heat for drying the clothes. In place of the steam being allowed to exhaust to the atmosphere, a special "back-pressure" valve is fitted in the exhaust pipe, and it is so arranged that the steam has sufficient pressure to force its way through a coil of pipes. Air is forced over the coil of pipes, being heated in the process, the warmed air being driven through the drying chamber and absorbing the water from the clothes that have been washed in the usual way.

The Drainage.

The drainage from the heating radiators, from steam pipes, from the steriliser, from every apparatus which uses steam, and in which steam is condensed into water, is led, by means of separate pipes, to a "manifold" header; a pipe which receives all the steam and all the hot water, and delivers them to either the receiver or the heating coils of the "Nuconomiser," an overflow pipe carrying off any surplus to the drains.

## Figures and Tables

**Figure f1:**